# In this month’s Bulletin

**DOI:** 10.2471/BLT.20.001220

**Published:** 2020-12-01

In editorials on antimicrobial resistance, Peter Beyer et al. (822) announce a new research and development fund and Timo Minssen et al. (823) examine social, cultural and economic aspects of antimicrobial overuse.

In the news section, Andréia Azevedo Soares (826–827) describes efforts to ensure equitable access to future vaccines for SARS-CoV-2. Cheryl Cohen talks to Gary Humphreys (828–829) about the response to the COVID-19 pandemic and surveillance for respiratory infections in South Africa.

## China 

### Keeping mild cases out of the community 

Juan Li et al. (830–841) detail the function of Fangcang shelter hospitals in Wuhan.

## Ethiopia

### Universal health coverage 

Yibeltal Assefa et al. (894–905) review evidence of primary health care impacts.

## Ghana

### Care of injured people 

Adam Gyedu et al. (869–877) study availability of health insurance and surgery.

## Republic of Korea

### Converting a university hospital for the red zone 

Mhinjine Kim et al. (842–848) describe the protocols used to make a dedicated COVID-19 hospital.

## South Africa

### A decade of data on body mass index

Muchiri E Wandai (878–885) establish trends in normal, overweight and obese categories. 

## United Republic of Tanzania

### Stars for better service 

Anna D Gage et al. (849–858) assess quality improvement measures in health facilities.

### Improving sickle cell surveillance

Emmanuela E Ambrose (859–868) re-use dried blood spots to measure prevalence of sickle cell trait and disease. 

## WHO Region of Africa

### Primary health-care gaps 

Azeb Tesema et al. (906–908) list changes needed to cover noncommunicable diseases.

## Global

### Improving nutrition

Christian Kraef et al. (886–893) focus on the cause of 1 in 5 global deaths. 

